# Massive Amniotic Fluid Aspiration in a Case of Sudden Neonatal Death With Severe Hypoplasia of the Retrotrapezoid/Parafacial Respiratory Group

**DOI:** 10.3389/fped.2019.00116

**Published:** 2019-04-04

**Authors:** Anna M. Lavezzi, Antonella Poloniato, Rosanna Rovelli, Laura Lorioli, Gabriela Alejandra Iasi, Teresa Pusiol, Graziano Barera, Stefano Ferrero

**Affiliations:** ^1^Department of Biomedical, Surgical and Dental Sciences, Lino Rossi Research Center for the Study and Prevention of Unexpected Perinatal Death and SIDS, University of Milan, Milan, Italy; ^2^Neonatal Unit, IRCCS Ospedale San Raffaele, Milan, Italy; ^3^Pathology Unit, IRCCS Ospedale San Raffaele, Milan, Italy; ^4^Institute of Pathology, Hospital of Rovereto, Rovereto, Italy; ^5^Division of Pathology, Fondazione IRCCS Ca' Granda, Ospedale Maggiore Policlinico, Milan, Italy

**Keywords:** newborn, sudden neonatal death, amniotic fluid inhalation, brainstem, retrotrapezoid/parafacial respiratory group, hypoplasia

## Abstract

We report a case of a baby, who, after pregnancy complicated by maternal Addison's disease and Hashimoto's thyroiditis and natural delivery, unexpectedly presented a cardiorespiratory collapse and died 1 hour after birth without responding to prolonged neonatal resuscitation maneuvers. The cause of death was reliably established by carrying out a forensic postmortem examination. More specifically, the histological examination of the lungs showed the presence of abundant endoalveolar and endobronchial cornea scales caused by absorption of amniotic fluid. The neuropathological examination of the brainstem highlighted severe hypodevelopment of the retrotrapezoid/parafacial respiratory group, which is a complex of neurons located in the caudal pons that is involved in respiratory rhythm coordination, especially expiration, in conditions of enhanced respiratory drive, as well as in chemoreception. This neuropathological finding shed new light on the mechanisms underlying the massive amniotic fluid aspiration which led to this early death.

## Introduction

Every year 2.7 million newborns die worldwide and more than 1 million newborns die on the first day of life, making the day of birth the most unsafe day for babies in nearly every country. According to the report published by Save the Children entitled “Ending Newborn Deaths” (1), the first few hours of postnatal life are definitely very dangerous as this is a critical time of transition from intra-uterine to extra-uterine life, when newborns are less responsive and more vulnerable to the outside world and stressors (2–4). Most of these newborn deaths are unexpected and inexplicable and therefore are defined as “early-SIDS” (5). The Italian law no.31 “*Regulations for Diagnostic Post Mortem Investigation in Victims of Sudden Infant Death Syndrome (SIDS) and Sudden Intrauterine Unexpected Death Syndrome (SIUDS)*,” which was passed in 2006, decrees that all infants who died suddenly in Italian regions within the first year of age, if SIDS is suspected, must undergo an in-depth autopsy including a thorough examination of the autonomic nervous system (6). This paper reports an interesting case of a newborn who after cord clamping experienced neonatal collapse and did never respond to neonatal resuscitation, for whom the application of the aforementioned guidelines enabled us to formulate a hypothesis on the pathogenic mechanism of death.

## Case Presentation

Following a 40-week pregnancy, uncomplicated labor and delivery, a female newborn unexpectedly showed cardiovascular and respiratory collapse immediately after cord clamping. Despite immediate resuscitation maneuvers prolonged for 1 hour she died. The newborn was well-developed, without visible malformations to the inspection. Birth weight was 3,270 g (adequate for gestational age at 45° percentile—IneS Charts), length was 48.5 cm (adequate for gestational age at 21° percentile—IneS Charts), and head circumference was 34 cm (46° percentile—IneS Charts) (7). Immediately after delivery, the neonate appeared pale, atonic, with absence of spontaneous reflexes and breathing. Apgar score was 1 at the 1st minute, 0 at the 5th and 10th minutes (1st minute, heart rate 60 bpm). Cord blood gases did not reveal metabolic acidosis. At the 1st minute, airways were aspirated in laryngoscopy with abundant emission of meconium stained liquid; thereafter the neonate was instantaneously intubated and positive pressure ventilation was started. In the absence of any clinical response, external chest compressions were promptly started. Indeed, the umbilical vein was incannulated with administration of adrenaline (three subsequent doses) and fluid loads. Resuscitation was performed according with the Neonatal Life Support criteria (8, 9). At the 50th minute, resuscitation was suspended due to the absolute absence of clinical response. In the absence of an identifiable cause of death, an initial diagnosis of suspected early-SIDS was made.

The 38-year-old mother was affected by Addison's disease and Hashimoto's thyroiditis and had been taking specific medication during pregnancy and labor time (as increase of corticosteroid therapy as foreseen by international guidelines). Precisely, the autoimmune thyroiditis was treated with Levo-tiroxina 100 mcg and the Addison disease with both Fludrocortisone acetate compress 0.2 mg/die and Cortone acetate compress 31.25 mg/die (every 8 hours during labour). As soon as the labor started, intravenous hydrocortisone 100 mg every 6 hours was continuously infused till the delivery. When questioned, the mother denied cigarette smoking and drug and alcohol consumption before and during pregnancy. However, the father was known for cigarette smoking during his wife's pregnancy.

In accordance with the directives of the aforementioned Italian law, a full post-mortem investigation including placenta and umbilical cord examination was performed within 48 h of death. The placenta, disc-shaped with well-analyzable maternal and fetal surfaces, weighed 500 g. Placental diameter ranged between 15 and 17 cm with a maximum thickness of 5 cm and a minimum of 3 cm.

The umbilical cord was inserted in the center of the fetal surface with well-defined blood vessels, which branched outwards and followed a twisted spiral course. It was 17 cm long with minimum and maximum diameters of 1 and 1.5 cm, respectively.

Multiple samples of all organs were collected at autopsy and fixed in 10% formalin buffer, processed and embedded in paraffin. Four-micrometer-thick (4 μm) sections were then cut from each sample and stained with hematoxylin/eosin for the histological examination.

### Histological Examination of the Organs

**Figure 1:**
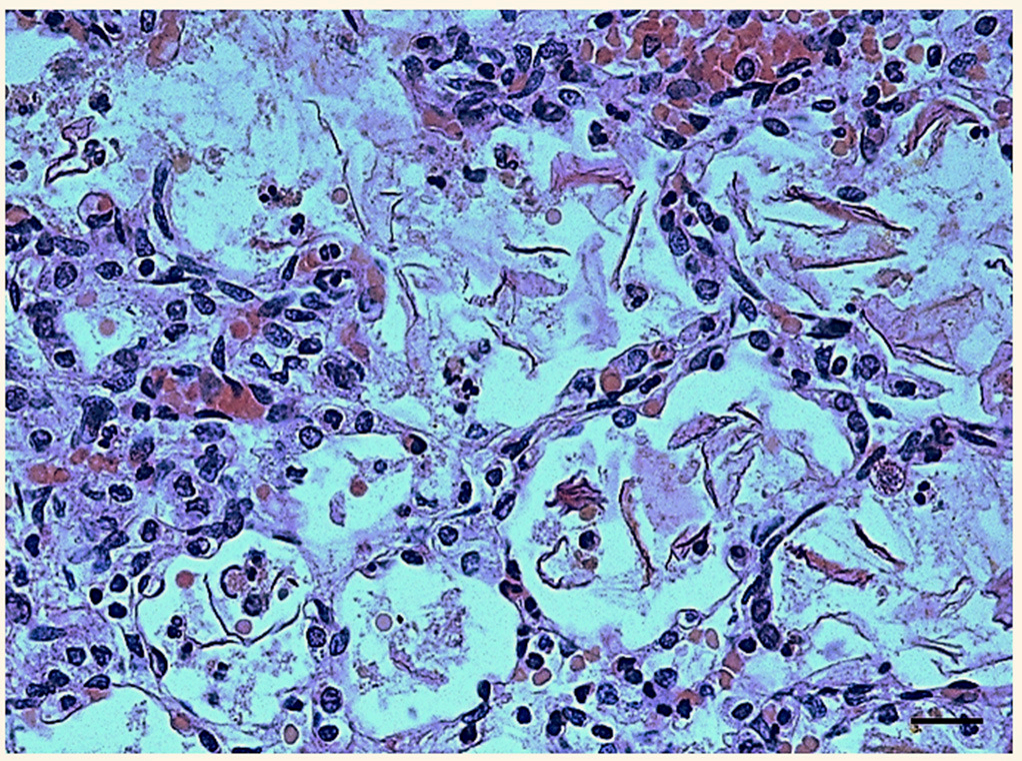
Histological section of lung showing the presence of abundant endoalveolar and endobronchial corneal scales into the alveoli. Staining: hematoxylin/eosin; Scale bar: 50 μm.

The lung sections highlighted the presence of abundant endoalveolar and endobronchial corneal scales induced by the aspiration of amniotic fluid, with meconium residues; several areas of emphysema, probably of a compensatory nature, were also detected especially in the right lung (Figure 1). No alterations were observed in the other organs at routine examination. Therefore, the anatomopathological diagnosis was that death was due to “severe pneumopathy caused by amniotic fluid aspiration.” At this point, presuming poor respiratory coordination at birth behind this finding, an in-depth study on the nervous system and especially the brainstem, which is where the main structures involved in breathing control are located, was carried out according to the guidelines given below.

### Neuropathological Examination of the Brainstem

**Figure 2:**
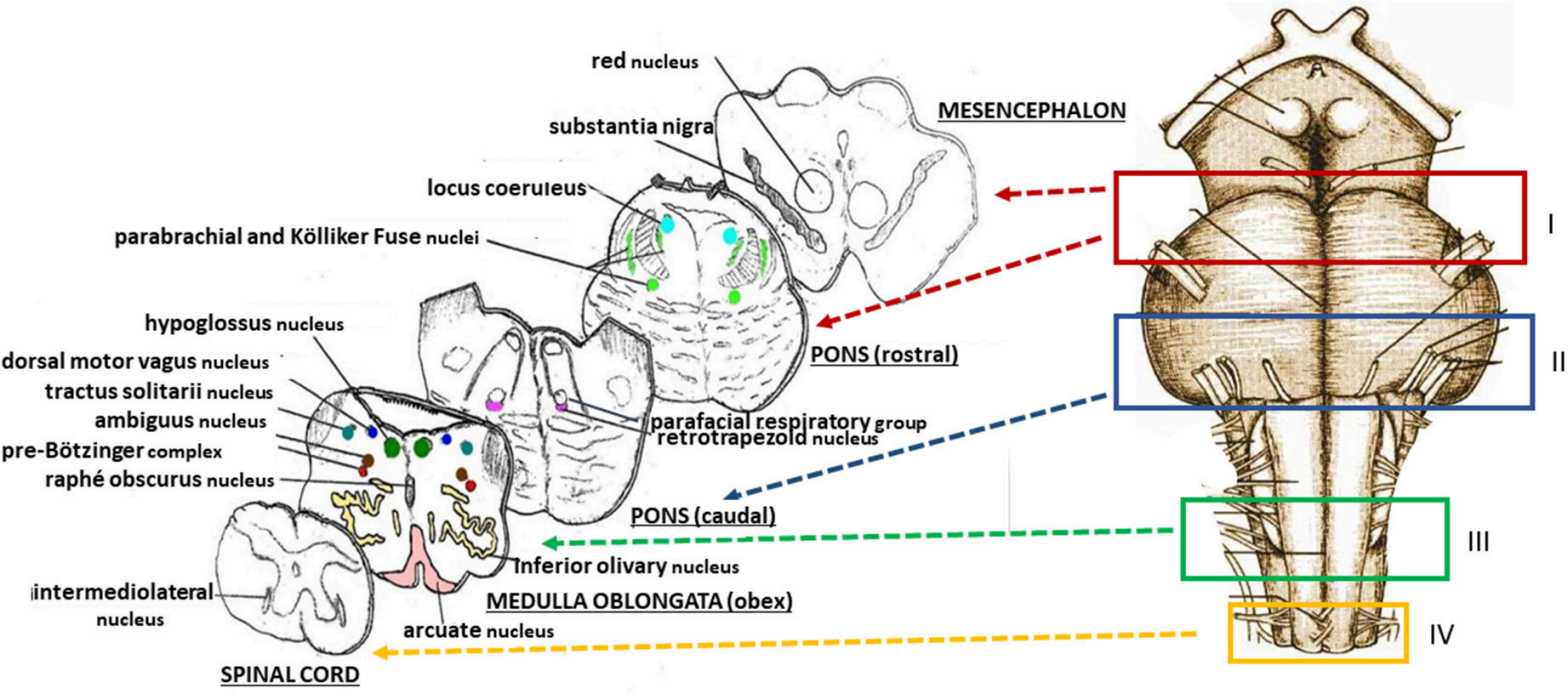
**(Right)** Schematic representation of the brainstem sampling: **I** = ponto-mesencephalic specimen, including the upper third of the pons and the adjacent portion of mesencephalon; **II** = caudal pontine specimen; **III** = medulla oblongata specimen, including the obex; **IV** = sample extending from the caudal pars of the medulla oblongata to the rostral spinal cord. **(Left)** schematic representative histological sections obtained from the four specimens, indicating the main nuclei and structures to be examined.

The protocol drawn up by the “Lino Rossi” Research Center of the Milan University in the context of the aforementioned Italian law, states that four specimens are to be taken from the brainstem after the routine fixation (as shown in Figure 2, at the right) (10). Transverse serial sections from all the samples are made at intervals of 60 μm. For each level, twelve 4 μm sections are obtained, two of which are routinely stained for histological examination using alternately hematoxylin-eosin and Klüver-Barrera stains. Additional sections are saved and stained as deemed necessary for specific immunohistochemical investigations. Figure 2, on the left, shows representative histological sections obtained from the above-described specimens, indicating the main nuclei and structures to be examined, given their frequent involvement in sudden perinatal deaths in terms of delayed development (hypoplasia/agenesis).

We have initially taken into account a possible analogy of the case reported here with the congenital central hypoventilation syndrome (CCHS). This is a disorder of respiratory and autonomic regulation usually manifesting in newborn period with apnea, hypoxemia, and hypercapnia, associated to a heterozygous mutation, in the form of polyalanine expansion, of the *PHOX2B* gene (11). Then, considering that PHOX2B expression is a specific feature of neurons that are postsynaptically CO_2_ sensitive in the retrotrapezoid nucleus (RTN) (12–15), we wanted to perform genetic and immunohistochemical analyses of this gene to highlight a possible mutation or defective expression which could account for the death.

#### PHOX2B Immunohistochemistry

To identify the cytoarchitecture of the RTN and its boundaries in the parafacial region, we applied the specific immunohistochemical technique to analyze the pattern of expression of PHOX2B using a rabbit polyclonal antibody raised against the 14 amino acid C-terminal sequence of the corresponding protein. Detailed procedure has been previously described (16). This analysis has been also extended to an age-matched case of death at birth for severe cardiomyopathy as control.

#### *PHOX2B* Genetic Analysis

Formalin-fixed, paraffin-embedded cerebral cortex samples, properly collected for the genetic testing, were primarily cut into 6 slices of 5 μm sections. Total DNA was extracted from this material and evaluated through PCR reaction, according to our previously published method (16).

### Results of the Neuropathological Examination of the Brainstem

**Figure 3:**
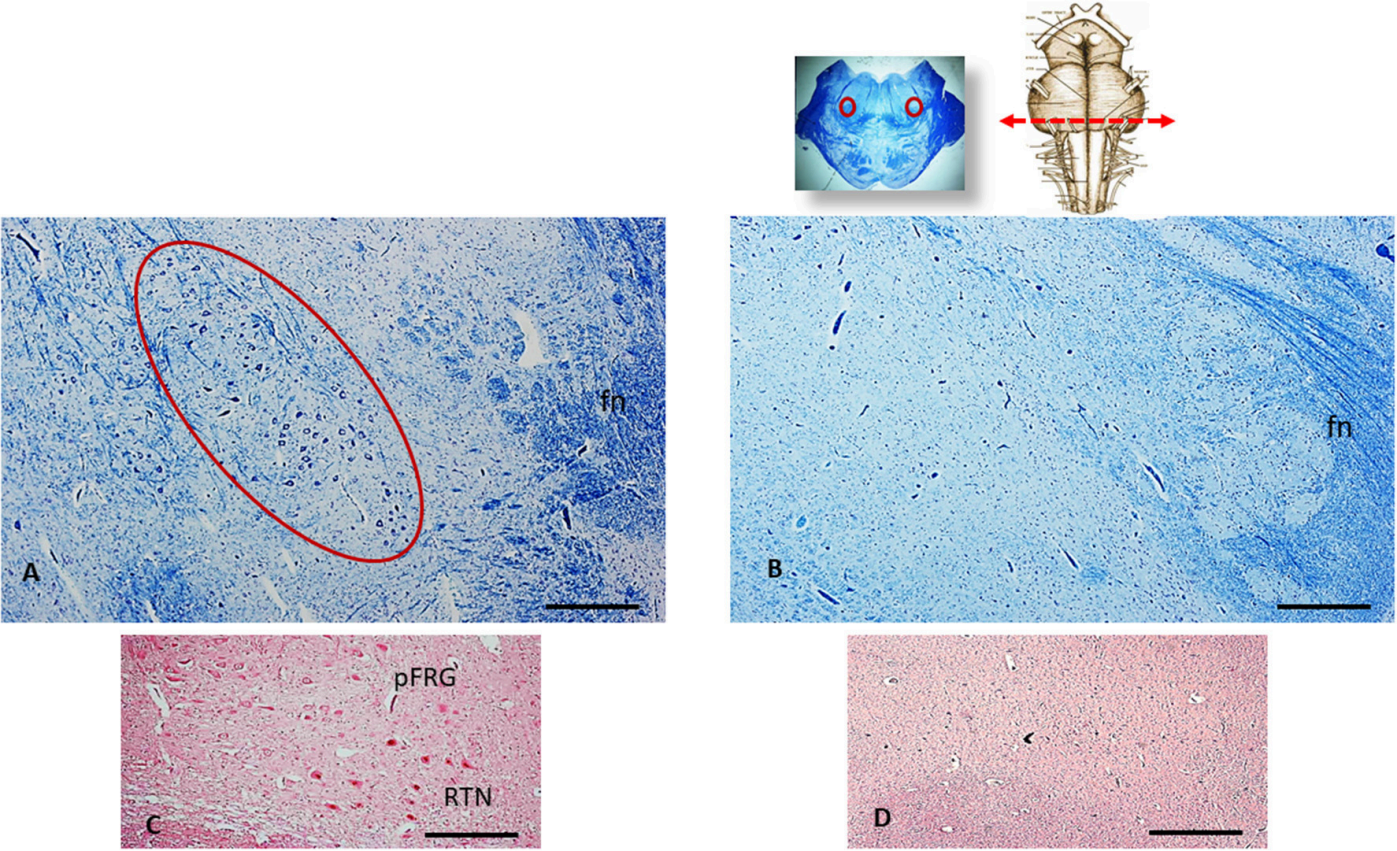
An image series related to the retrotrapezoid/parafacial respiratory group (RTN/pFRG). At the top, on the right: ventral brainstem schematic image with the indication (red arrow) of the optimal sampling for the examination of the human RTN/pFRG in the caudal pons; on the left: whole histological section at this level with the indication of the RTN/pFRG localization. **(A)** Histological section of medulla oblongata showing in the circled area the normal cytoarchitecture of the RTN/pFRG, adjacent to the facial nerve (fn) in an age-matched control case. **(B)** Severe hypoplasia of the RTN/pFRG observed in this case. **(C)** Cluster of PHOX2B immunoreactive neurons identifier of the RTN in the control case. **(D)** Lack of PHOX2B immunoreactivity in the RTN region. **(A,B)** Staining: Klüver-Barrera; **(C,D)** Staining: PHOX2B immunohistochemistry. Scale bar **(A–D)**: 100 μm.

The histological analysis of the brainstem showed severe hypoplasia of the retrotrapezoid/parafacial respiratory group (RTN/pFRG) in the caudal pons when compared with the normal structure of this complex in the control case (Figures 3A,B). The agenesis of the RTN, in particular, was supported by the immunohistochemical detection of the PHOX2B gene expression. Intensely PHOX2B immunoreactive nuclei were present in fact only in the neurons of the control case, allowing to easily distinguish the RTN from the overlying parafacial neurons (Figure 3C), while no PHOX2B immunoreactivity was highlighted in the RTN region of the case object of this exposure (Figure 3D).

**Figure 4:**
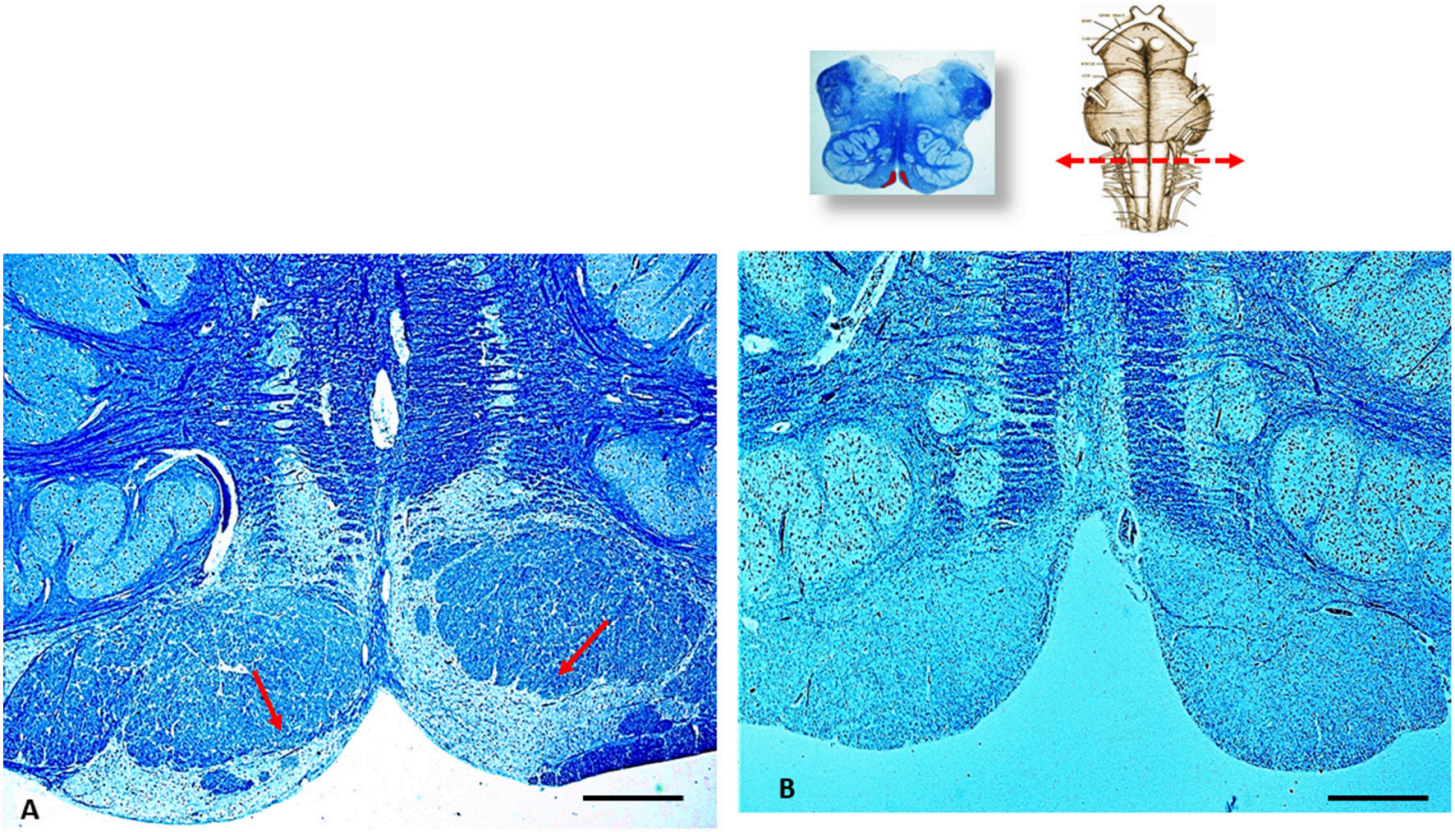
An image series related to the arcuate nucleus (AN). At the top, on the right: ventral brainstem schematic image with the indication (red arrow) of the optimal sampling for the examination of the human AN in the medulla oblongata (at the obex level); on the left: whole histological section at this level with the indication of the AN localization. **(A)** Histological section showing (red arrows) the normal structure of the AN at the ventral medullary surface in an age-matched control case. **(B)** Severe hypoplasia of the AN observed in this case. **(A,B)** staining: Klüver–Barrera; Scale bar **(A,B)**: 100 μm.

In addition, a severe hypoplasia of the arcuate nucleus in the medulla oblongata was observed (Figure 4). All the other main structures (represented in Figure 2, on the left) showed a normal cytoarchitecture, including the Kölliker-Fuse nucleus (KFN) and the pre-Bötzinger complex (preBötC), both determinant neuron groups for respiratory pattern generation.

### Result of the *PHOX2B* Genetic Analysis

The genetic analysis performed on DNA extracted from the cerebral cortex showed a normal *PHOX2B* 20/20 genotype without evidence of a polyalanine repeat expansion mutation involving the second polyalanine repeat sequence in exon 3.

## Discussion

The case of a newborn born healthy but who died inexplicably after delivery is reported. At autopsy, a massive presence of amniotic fluid in the lungs was declared to be the primary cause of death. Following a thorough examination of the autonomic nervous system, this pathological finding was associated to a specific brainstem alteration, more specifically a developmental defect of the RTN/pFRG, which is a neuronal center located in the caudal pons involved in respiratory pattern.

Breathing is an essential behavior which must be able to function perfectly at the end of pregnancy (17). At delivery, the lungs are still full of fluid until the first postnatal breath is taken. The first breath is characterized by the rapid transition from fluid- to air-filled lungs (18–20). In particular, the first inspiratory effort plays an essential role by generating an active pressure gradient which shifts the fluid into the interstitial tissue, where it is gradually removed by the pulmonary and lymphatic circulations. In humans, the pharynx serves as common pathway for both breathing and swallowing, two processes closely interrelated (21, 22). Swallowing momentarily inhibits breathing and the resulted apnea is followed by expiration. Then, breathing and swallowing do not occur simultaneously but their functions are mutually exclusive to protect from aspiration. Precisely, at the end of the first deep inspiration, which is also supported by the contraction of the diaphragm, the glottis closes to prevent the conveyance of foreign substances into the respiratory tract and to maintain the lungs full of air, avoid gas loss and facilitate prolonged expiration. Neurological alterations can cause breakdown of the normal breathing pattern, as prolonged swallow apneas are followed by inspiration (23). The initiation of breathing and the coordination of the respiratory rhythmic pattern under conditions of normal or eupneic breathing comprises three phases: active inspiration, post-inspiration (corresponding to an inspiratory pause) and active expiration (24). These phases are essential for oxygen supply to tissues and carbon dioxide removal (25).

Control of the timing of the inspiratory/expiratory (IE) phase transition is regulated by the autonomic nervous system. Under physiological conditions, the alternating phases of inspiration and expiration are largely governed by ponto-medullary interaction between distinct inspiratory and expiratory neurons (26–28). Janczewski and Feldman (29) reported the existence of two separate, functionally distinct rhythm generators in the brainstem for inspiration and active expiration, respectively, which originated in two different rhombomeres. By carrying out inspiratory and expiratory studies on mouse brain transections, these authors identified the pre-BötC in the medulla oblongata as a dominant site for inspiration rhythm generation, and the RTN/pFRG, consisting of two adjacent and sometimes overlapped neuronal centers in the caudal pons, located ventral to the facial motor nucleus, as active expiratory center. However, this view seems contradictory to the subsequent findings obtained by various groups of researches. According to the experimental studies of Onimaru et al. (14, 30, 31), the pFRG is predominantly composed of pre-inspiratory neurons which are hypothesized to trigger onset of bursting in the effective inspiratory center, the preBötC. These authors support the concept that coupling between the pFRG and preBötC is important in the generation of the primary respiratory rhythm at birth. Now, it is well-accepted that the pFRG is a conditional expiratory oscillator that is actively inhibited during restful eupnea by the KFN that is responsible of the I/E phase transition control. In eupnea, the Bötzinger complex (BötC), that is adjacent to the more caudal pre-BötC, is the true expiratory half-center that provides inhibition of the phase-switch, determining the length of exhalation whilst integrating drive from mechano- chemoreceptors and pontine centers (32–35). Under conditions of elevated metabolic demand, such as hypercapnia or hypoxia, the pFRG is activated because of increased excitation from central and peripheral receptors, resulting in active exhalation to support the required increased pulmonary ventilation (36). Accumulated evidence indicates that the subgroup of neurons of the RTN, located close to the pFRG, express the transcription factor *PHOX2B* and plays an important role in chemosensory integration, including central CO_2_ chemoreception in respiratory control (12, 13, 37), so providing drive to both inspiratory and expiratory neuronal populations. The RTN neurons are easily excitable under hypercapnic conditions, contributing to the dynamic control of the acid-base status by regulating ventilatory activity (38, 39). Very recently, Zoccal et al. (40) demonstrated that excitatory inputs from chemosensitive neurons in the RTN are necessary for the activation of the expiratory neurons in the pFRG during hypercapnia, showing that these two centers are constituted by functionally and phenotypically distinct but synaptically interacting populations. The RTN-*PHOX2B* neurons establish also direct excitatory glutamatergic synapses with the pre-BötC inspiratory neurons, so making an important contribution to the respiratory rhythm-generating circuitry (41).

The current understanding of the physiology and function of the RTN/pFRG and of the other components of the respiratory network, has at this moment been demonstrated in animal models. The role of these structures in breathing control in humans has yet to be investigated. Therefore, we can only hypothesize similar trends for humans, although we are aware that there may be differences between species (42).

However, our neuropathological findings were consistent with this hypothesis. The sudden neonatal death reported here could be related to the inhibition of the active exhalation burst that should have happened in the undoubted elevated respiratory need, as the alveoli were occluded by abundant amniotic fluid. In our opinion, although the KFN and the pre-BötC were normally developed, their input to the RTN/pFRG could not be implemented. The hypodevelopment even of only one center of the respiratory network can then compromise the functionality of the other components and then the total respiratory rhythmic pattern.

One last observation concerns the observation of arcuate nucleus hypodevelopment in the medulla oblongata. This alteration may have worsened the effect of the RTN/pFRG hypoplasia. Like the RTN, in fact, this nucleus is a chemosensitive structure involved in ventilator drive, whose main task is to maintain O_2_ and CO_2_ homeostasis in blood and tissues (43–45). Furthermore, the hypoplasia of the arcuate nucleus, resulting in a reduced response to increased CO_2_ levels, has been frequently implicated in sudden unexpected perinatal deaths (46, 47).

## Concluding Remarks

The case presented here emphasizes the fact that only an in-depth histopathological examination of the brainstem can give us a better understanding of the underlying pathogenetic mechanism of a sudden unexpected infant death. This report stresses the importance of applying the Italian law 31 worldwide, especially when a newborn dies suddenly during the first hours of life.

## Ethics Statement

Permission from the Ethics Committee and parent's consent were not required for this study as the Lino Rossi Research Center of the Milan University is the national referral center for the application of the aforementioned Italian Law n. 31 on fetal and infant death. Study approval was anyway granted by the institutional review board of Milan University.

## Author Contributions

AL was responsible for the neuropathological examination and the study planning. AP and LL actively contributed to the clinical management of the patient and manuscript writing. RR contributed to the clinical management of the patient. GB supervised manuscript writing and clinical management. GI performed the autopsy and post-mortem evaluations. TP participated in the interpretation of the neuropathological results and reviewed the English language. SF gave collaborative input for the drafting of the article.

### Conflict of Interest Statement

The authors declare that the research was conducted in the absence of any commercial or financial relationships that could be construed as a potential conflict of interest.
